# Methods to Adjust for Confounding in Test-Negative Design COVID-19 Effectiveness Studies: Simulation Study

**DOI:** 10.2196/58981

**Published:** 2025-01-27

**Authors:** Elizabeth AK Rowley, Patrick K Mitchell, Duck-Hye Yang, Ned Lewis, Brian E Dixon, Gabriela Vazquez-Benitez, William F Fadel, Inih J Essien, Allison L Naleway, Edward Stenehjem, Toan C Ong, Manjusha Gaglani, Karthik Natarajan, Peter Embi, Ryan E Wiegand, Ruth Link-Gelles, Mark W Tenforde, Bruce Fireman

**Affiliations:** 1 Westat Rockville, MD United States; 2 Vaccine Study Center Northern California Division of Research Kaiser Permanente Oakland, CA United States; 3 Center for Biomedical Informatics Regenstrief Institute Indianapolis, IN United States; 4 Fairbanks School of Public Health Indiana University Indianapolis, IN United States; 5 HealthPartners Institute Minneapolis, MN United States; 6 Center for Health Research Kaiser Permanente Portland, OR United States; 7 Division of Infectious Diseases and Clinical Epidemiology Intermountain Healthcare Salt Lake City, UT United States; 8 Department of Biomedical Informatics University of Colorado Anschutz Medical Campus Aurora, CO United States; 9 Department of Pediatrics Section of Pediatric Infectious Diseases Baylor Scott & White Health Temple, TX United States; 10 Department of Medical Education Texas A&M University College of Medicine Killeen, TX United States; 11 Department of Biomedical Informatics Columbia University Irving Medical Center New York, NY United States; 12 New York Presbyterian Hospital New York, NY United States; 13 Vanderbilt University Medical Center Nashville, TN United States; 14 National Center for Immunization and Respiratory Diseases Centers for Disease Control and Prevention Atlanta, GA United States

**Keywords:** disease risk score, propensity score, vaccine effectiveness, COVID-19, simulation study, usefulness, comorbidity, assessment

## Abstract

**Background:**

Real-world COVID-19 vaccine effectiveness (VE) studies are investigating exposures of increasing complexity accounting for time since vaccination. These studies require methods that adjust for the confounding that arises when morbidities and demographics are associated with vaccination and the risk of outcome events. Methods based on propensity scores (PS) are well-suited to this when the exposure is dichotomous, but present challenges when the exposure is multinomial.

**Objective:**

This simulation study aimed to investigate alternative methods to adjust for confounding in VE studies that have a test-negative design.

**Methods:**

Adjustment for a disease risk score (DRS) is compared with multivariable logistic regression. Both stratification on the DRS and direct covariate adjustment of the DRS are examined. Multivariable logistic regression with all the covariates and with a limited subset of key covariates is considered. The performance of VE estimators is evaluated across a multinomial vaccination exposure in simulated datasets.

**Results:**

Bias in VE estimates from multivariable models ranged from –5.3% to 6.1% across 4 levels of vaccination. Standard errors of VE estimates were unbiased, and 95% coverage probabilities were attained in most scenarios. The lowest coverage in the multivariable scenarios was 93.7% (95% CI 92.2%-95.2%) and occurred in the multivariable model with key covariates, while the highest coverage in the multivariable scenarios was 95.3% (95% CI 94.0%-96.6%) and occurred in the multivariable model with all covariates. Bias in VE estimates from DRS-adjusted models was low, ranging from –2.2% to 4.2%. However, the DRS-adjusted models underestimated the standard errors of VE estimates, with coverage sometimes below the 95% level. The lowest coverage in the DRS scenarios was 87.8% (95% CI 85.8%-89.8%) and occurred in the direct adjustment for the DRS model. The highest coverage in the DRS scenarios was 94.8% (95% CI 93.4%-96.2%) and occurred in the model that stratified on DRS. Although variation in the performance of VE estimates occurred across modeling strategies, variation in performance was also present across exposure groups.

**Conclusions:**

Overall, models using a DRS to adjust for confounding performed adequately but not as well as the multivariable models that adjusted for covariates individually.

## Introduction

### Background

Real-world, observational vaccine effectiveness (VE) studies are critical for evaluating the post licensure performance of vaccines. These studies must address confounding from patients’ demographic characteristics and underlying medical conditions because such factors may be associated with both disease outcomes and vaccination status. When vaccination status was dichotomous, inverse probability weighting based on propensity scores (PS) could be used to adjust for confounding. However, the usefulness of the PS is problematic when the exposure is multinomial rather than dichotomous—for example, if we are interested in comparing multiple levels of vaccination status distinguished by the number of doses received and by intervals of time after the last dose [1]. Multinomial PS approaches are feasible in some situations [2], however, multinomial PS poses computational challenges and is less intuitive.

An alternative to the PS in this context is the disease risk score (DRS), also called a confounder score, prognostic score, comorbidity score, or simply a risk score [1-9]. A DRS can combine covariates into a single score that reflects their associations with the outcome. However, if it is feasible to make a DRS that adjusts appropriately for the relevant covariates, it can be similarly feasible and appropriate to simply adjust for the covariates individually without first combining them into a DRS [1]. This simulation study compared logistic regression models that use a DRS to adjust VE estimates versus logistic regression models that adjust for covariates individually. We compared DRS adjustment with individual covariate adjustment in scenarios comprised of simulated data similar to real-world data used by the Virtual SARS-CoV-2, Influenza, and Other Respiratory Viruses Network (VISION) to report on COVID-19 VE.

### VISION: Virtual SARS-CoV-2, Influenza, and Other Respiratory Viruses Network Studies of COVID-19 VE

The VISION network was established by the Centers for Disease Control and Prevention (CDC) in collaboration with 10 US health care systems with medical, laboratory, and vaccination records. VISION uses a case-control test-negative design (TND) to assess the effectiveness of COVID-19 vaccines in preventing laboratory-confirmed COVID-19–associated hospitalizations and visits to emergency departments or urgent care clinics [10]. Patients who received care in one of these settings for a COVID-19-like illness (CLI) are included in the study if they were tested for SARS-CoV-2 by molecular assay proximate to the encounter. Those who tested positive were considered cases; those who tested negative were considered controls. CLI diagnoses include acute respiratory diagnoses or related signs or symptoms, captured by diagnosis codes [10-13]. The case-control TND has been commonly used in studies of COVID-19 VE and VE studies against influenza, rotavirus, and other diseases [14-16]. This study was reviewed and approved by the institutional review board at Westat, Inc. This electronic health record-based study does not include factors necessitating patient consent. The findings and conclusions in this report are those of the authors and do not necessarily represent the official position of the CDC.

When VISION first began analyzing COVID-19 VE in early 2021, the comparison of interest was between fully vaccinated individuals and unvaccinated individuals. PS-based methods have been widely used in cohort studies with this kind of binary exposure [17-19]. VISION’s case-control studies also used a PS, derived from the test-negative controls, to adjust VE estimates [10,20].

As the pandemic progressed, recommended vaccination schedules became more complex [21-23]. Research studies required consideration of more nuanced exposure categories, based on a combination of the number of vaccine doses received and time since the most recent vaccine dose (to examine the waning of vaccine-induced protection against COVID-19) [24-26]. VISION continued to rely on the PS for covariate adjustment by conducting a series of separate comparisons, each comparing one vaccine status with another. For example, 1 PS was derived and used in comparisons of vaccinees who received 3 doses versus the unvaccinated; then a separate PS was derived and used in comparisons of vaccinees who received 4 doses versus the unvaccinated. However, separate models do not allow for easy testing between different levels of exposure and are computationally challenging, with a single analysis often requiring over 1000 PS to account for different vaccination exposure groups and population subgroups being assessed. In addition, the positivity assumption of the PS requires that at no level of the PS is outcome probability close to 0 or 1 [27,28]. The widening difference in characteristics between those who closely follow the recommended vaccination schedule and those remaining unvaccinated put a strain on the positivity assumption and decreased the appeal of collapsing vaccination categories into two.

### Disease Risk Scores to Adjust for Confounding

An alternative to PS is the DRS. A DRS reflects the relationship between potential confounders and the outcome [1-4,7,9,29,30]. Conditioning on a DRS does not balance covariates across treatment groups as a PS would but results in a covariate balance where the potential outcome under the referent condition is independent of a set of covariates [29], which has been called prognostic balance by Hansen [30]. In short, the DRS is an observation’s predicted risk of the outcome, assuming the observation was in the referent category of the exposure, reflecting the risk of the outcome relative to other observations. There are well-known generalizable risk scores, such as the Gail model score [31], Framingham score [32], APACHE score [33], and Charlson index [34] that can be used to control for baseline health status. Instead, a study may generate a DRS specific to the outcome and population. In the context of a TND, the outcome is not simply testing positive for the disease of interest but rather testing positive for the disease of interest conditional on receiving care and being tested. A key benefit of using the DRS is potentially only calculating it once for the entire dataset, or a few times for a few key subgroups, rather than generating up to thousands of PS for comparisons using different dichotomous comparisons between multiple vaccination exposure groups.

We compared the performance of DRS-adjusted estimators with multivariable-adjusted estimators that do not aggregate the covariates into a composite score. Several studies have implemented TNDs with this straightforward approach—that is, by fitting logistic regression models that include many covariates in addition to indicators of vaccination status [35-37]. While many analytic methods select variables or adjust for confounding, we are particularly interested in the possibility the DRS allows us to calculate a single score to apply to many analyses.

## Methods

### Monte Carlo Simulation

We conducted simulations to compare the performance of alternative approaches to the adjustment of VE estimates for potential confounding—either by DRS or by individual covariate adjustment—in VISION network-like scenarios. In order of generation, the simulated data consisted of a bootstrapped sample of individuals, each with (1) a profile of continuous and categorical covariates, (2) a 13-level vaccination status derived conditionally from the covariates, and (3) a 2-level outcome derived conditionally from vaccination status and the covariates. The simulation study used *R*=1000 replications, which ensured that an estimated coverage of 95% would have a Monte Carlo error of 0.7 [38]. Datasets were generated as follows:

1. A bootstrap sample of size N=1000 or 10,000 observations was drawn from observed emergency department and urgent care encounters in VISION data of adults aged ≥18 years from the Omicron predominance era (December 16, 2021-July 31, 2022). Each observation consisted of all covariates aside from vaccination status and test result for a sampled encounter. This maintained complex relationships between potential confounders in the real-world VISION study. The data used to initiate this simulation study were accessed beginning on August 8, 2022. The authors did not have access to information that could identify individual participants during or after the analysis.
2. Vaccination status was generated as a 13-level exposure variable incorporating the number of vaccine doses received and the time interval since receipt of the most recent dose from a multinomial distribution for each observation, *V_i_*, based on the bootstrapped covariates, *Xi* (1).

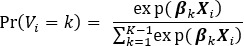
, where *k*=1,2,…,13 and *i*=1,2,…, N (1)

***β****_k_* was derived from the dataset by multinomial logistic regression and shown in Table S1 in Multimedia Appendix 1. The generated 13-level vaccination status was collapsed into a 5-level vaccination status as shown in Table 1 with approximate frequencies. We focused on VE estimates for each of the 4 levels of vaccination status compared with a reference level of vaccination status.

Next, we used a Bernoulli distribution to generate an outcome, *Y_i_* dependent on the covariates in the bootstrap sample, and generated 13-level vaccination status (2). **γ** are the coefficients describing the true VE and are shown in Table 1. ***α*** are defined in Table S2 in Multimedia Appendix 2. In the VISION data, from which the bootstrap sample was drawn, approximately 22% of encounters were cases. This percentage varied depending on covariates such as season or vaccination status. For example, cases comprise approximately 42% of encounters in January 2022 and only 13% of encounters in May. Among those unvaccinated, approximately 30% are cases, while among those recently vaccinated with their third dose, 11% are cases.



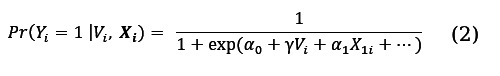



**Table 1:** Definition of vaccination status categories for data generation and analysis with the true VE used in generating simulated data.^a^

Thirteen-level vaccination status categories for data generation				Five-level vaccination status categories for VE analysis	
Definition^b^	Approximate frequency in simulated data^c,d^, %	Coefficient for true VE, γ	Corresponding true VE	Definition	Approximate frequency in simulated data^c,d^, %
Unvaccinated	40	—^e^	—	Unvaccinated	40
2 doses, 14-59 d	1	–1.05	65	2 doses, 14-179 d	6
2 doses, 60-119 d	2	–0.60	45	—	—
2 doses, 120-179 d	3	–0.43	35	—	—
2 doses, 180-239 d	5	–0.36	30	2 doses, ≥180 d	24
2 doses, 240-299 d	7	–0.29	25	—	—
2 doses, 300-359 d	6	–0.22	20	—	—
2 doses, ≥360 d	6	–0.16	15	—	—
3 doses, 7-59 d	6	–1.61	80	3 doses, 7-119 d	15
3 doses, 60-119 d	10	–1.39	75	—	—
3 doses, 120-179 d	8	–0.43	35	3 doses, ≥120 d	15
3 doses, 180-239 d	5	–0.16	15	—	—
3 doses, ≥240 d	1	–0.11	10	—	—

^a^VE: vaccine effectiveness.

^b^Vaccination categories are defined by the number of mRNA vaccines received at the index date of the medical encounter and the days between the most recent dose and the index date. The index date is the earlier of the date of specimen collection of a positive test within the allowable testing window (14 d before to 72 h after the encounter) and the time of the medical encounter.

^c^Approximate percent observed in the full dataset from which samples of 10,000 or 1000 are drawn.

^d^Data were generated using a 13-level vaccination status variable, with the approximate frequency of each category shown. This frequency varies across the 1000 replicates. The vector of coefficients, **γ**, is used as part of the data generation process, and its corresponding values on the VE scale are shown. In the analysis phase, the 13 categories are collapsed to the 5 shown in the column on the right, again with approximate frequencies.

^e^Not available.

### Derivation of the DRS

Methods for deriving the DRS have been described elsewhere [1-5,7,29,39]. For this simulation study, we used Miettinen’s approach, which models the outcome in relation to the covariates in the entire study population and includes the exposure of the vaccination status in the model with the covariates. The coefficients from the fitted model were used to calculate a predicted DRS for the entire dataset, predicting what risk of the outcome would be for each individual if they were unvaccinated (or if they were given the “reference” level of vaccination status in other scenarios) [5]. Multimedia Appendix 3 [40,41] contains more details about why this approach was selected.

Consideration around the use of a single DRS for the entire VISION study population for each analysis of VE was required. For example, VISION analyses were commonly stratified by age and immunocompromising status. Those with immunocompromising conditions had different vaccine recommendations and may have had reduced VE [42]. Accordingly, in each generated dataset, up to 3 DRS were estimated for each individual depending on their age and immunocompromised (IC) status. One used the full generated dataset, the second used only those with IC conditions according to VISION network criteria, and the third used only those 50 years and older (≥50).

In Miettinen’s approach, incorrectly modeling the modification of exposure by baseline covariates can result in estimated scores that are influenced by the magnitude of the exposure effect [30]. Applied to the estimation of DRS, gradient-boosted regression trees look for multilevel interactions that are useful for describing the relationship between patient characteristics, including vaccination status, and the outcome [20]. DRS were estimated using gradient boosted regression and included the exposure and all the covariates. Table S1 in Multimedia Appendix 1 contains a listing of these covariates.

### Final VE Models

The VE was calculated in a variety of logistic regression models, calculated as (1–adjusted odds ratio)×100%. Without a DRS, we estimated unadjusted VE, VE adjusted only for the key variables of patient age (as a spline), calendar date from January 1, 2021 (epi-day, as a spline), and VISION site and sub-region of the health care facility (site-region) (referred to as multivariable key), and VE adjusted for the key variables plus all other variables in the DRS (referred to as multivariable all). Note that the term spline refers to a natural cubic spline with 3 knots placed at the 25th, 50th, and 75th percentiles.

While a PS is typically used to balance comparisons of the exposed versus the unexposed by inverse probability weighting or by matching on the PS [43], there is less theory or experience pertaining to the use of the DRS in the final model that yields the adjusted VE [2]. A model may include DRS as a covariate [44], or a model could be conditional on DRS [45], which under certain conditions, may yield an average of the individual treatment effects of those treated (ATT) [29]. Well-known generalizable risk scores, such as the Gail model score, Framingham score, APACHE score, and Charlson index, are sometimes included in models or are used for stratification. The risk score we investigated was not designed to be generalizable, however, the modeling strategies employed for including a DRS in a VE model are similar. We investigated DRS inclusion in VE models as strata in a conditional model as well as a continuous covariate fit with a flexible spline. Conditional models used DRS as centiles, or deciles with epi-week, and site as strata. Conditional models that are only conditioned on DRS and models that included DRS as a continuous covariate also adjusted for age (as a spline), epi-day (as a spline), and site region. Models that were conditioned on DRS epi-week and site were also adjusted for age (as a spline). Vaccination status in the final VE model was collapsed to the 5-level category. Table 2 summarizes the calculated models.

**Table 2:** Description of final vaccine effectiveness (VE) models assessed in the simulation study.

Model name	Subset of data used to estimate DRS^a,b^. Subset of data used for estimating VE^c^.	Model stratification and adjustment	Dataset totals, n
Unadjusted	DRS dataset: no DRS. VE dataset: full simulated dataset.	Vaccination status only	10,000 and 1000
Multivariable all	DRS dataset: no DRS. VE dataset: full simulated dataset.	Stratification: none Adjustment: variables that would go into the DRS including age (spline), epi-day (spline), and site region	10,000 and 1000
Multivariable key	DRS dataset: no DRS. VE dataset: full simulated dataset.	Stratification: none Adjustment: age (spline), epi-day (spline), and site region	10,000 and 1000
Stratified week, site, DRS	DRS dataset: full simulated dataset. VE dataset: full simulated dataset.	Stratification: DRS decile, epi-week, and site Adjustment: age (spline)	10,000 and 1000
Stratified DRS	DRS dataset: full simulated dataset. VE dataset: full simulated dataset.	Stratification: DRS centile Adjustment: age (spline), epi-day (spline), and site region	10,000 and 1000
Spline DRS	DRS dataset: full simulated dataset. VE dataset: full simulated dataset.	Stratification: none Adjustment: DRS included with a natural spline with 4 degrees of freedom, age (spline), epi-day (spline), and site-region	10,000 and 1000
Multivariable key IC^d^	DRS datasets: no DRS. VE dataset: IC subset. DRS datasets: IC subset. VE dataset: IC subset.	Stratification: none Adjustment: age (spline), epi-day (spline), and site	10,000
Stratified week, site, IC DRS	DRS datasets: full simulated dataset. VE dataset: IC subset. DRS datasets: IC subset. VE dataset: IC subset.	Stratification: DRS decile, epi-week, and site Adjustment: age (spline)	10,000
Stratified IC DRS	DRS datasets: full simulated dataset. VE dataset: IC subset. DRS datasets: IC subset. VE dataset: IC subset.	Stratification: DRS centile Adjustment: age (spline), epi-day (spline), and site	10,000
Spline IC DRS	DRS datasets: full simulated dataset. VE dataset: IC subset. DRS datasets: IC subset. VE dataset: IC subset.	Stratification: none Adjustment: DRS included with a natural spline with 4 degrees of freedom, age (spline), epi-day (spline), and site	10,000
Multivariable key ≥50^e^	DRS datasets: no DRS. VE dataset: ≥50 subset. DRS datasets: ≥50 subset. VE dataset: ≥50 subset.	Stratification: none Adjustment: only for age (spline), epi-day (spline), and site region	10,000
Stratified week, site, ≥50 DRS	DRS datasets: full simulated dataset. VE dataset: ≥50 subset. DRS datasets: ≥50 subset. VE dataset: ≥50 subset.	Stratification: DRS decile, epi-week, and site Adjustment: age (spline)	10,000
Stratified ≥50 DRS	DRS datasets: full simulated dataset. VE dataset: ≥50 subset DRS datasets: ≥50 subset. VE dataset: ≥50 subset.	Stratification: DRS centile Adjustment: age (spline), epi-day (spline), and site region	10,000
Spline ≥50	DRS datasets: full simulated dataset. VE dataset: ≥50 subset. DRS datasets: ≥50 subset. VE dataset: ≥50 subset.	Stratification: none Adjustment: DRS included with a natural spline with 4 degrees of freedom, age (spline), epi-day (spline), and site region	10,000

^a^DRS: disease risk score.

^b^One goal of this simulation study is to consider how the DRS might behave in smaller subsets of the full dataset, specifically a subset of patients with IC conditions and a subset of patients 50 years or older. For this reason, when analyzing these subsets, we consider a DRS built from the full dataset but applied to a smaller subset and a DRS built specifically from that smaller subset.

^c^VE: vaccine effectiveness.

^d^IC: patients with immunocompromising conditions.

^e^≥50: patients aged 50 years or older.

### Assessing Model Performance

To evaluate performance, we examined bias, SE, the ratio of mean SE to empirical SE, and coverage. The true VE is built into the data generation mechanism, and these known values are shown in Table 1 for the 13 levels of vaccination status. However, VE was estimated for the collapsed 5-level vaccination status, *Vcollapsed*_i_. Therefore, for each replication, *r*, the true effect for each 5-level vaccination status, *γ_m,r_*, was approximated with a weighted average (weighted by sample size in each of the 13 vaccination status categories) as shown below (3) and summarized as a VE (4).



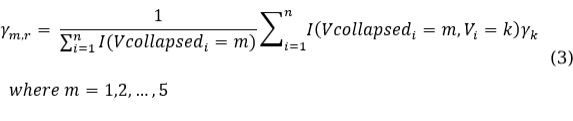





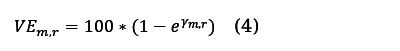



The estimated VE for each vaccine status in each replication, 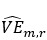, was calculated as shown in (5).



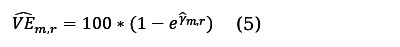



Percent VE bias was calculated as the average percent bias across the replications on the VE scale (6).



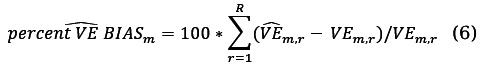



The ratio of the observed SE of the OR compared with the empirical SE was calculated as shown in (7).



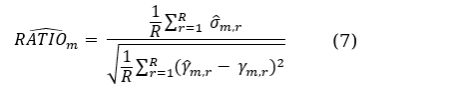



### Presentation of Results

Simulation results are presented in figures depicting the complete distribution of observed percent VE bias and SE of *γ_m,r_*, 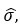 for each vaccination status. The average across all replications is shown as well as the 2.5th and 97.5th percentiles. The empirical SE, or SD of the coefficients, is presented as a vertical bar. In addition to low bias, an ideal model will provide estimates of SE that are also averaged across replications, close to the SD of the estimated coefficients. The figures include coverage. All results are in Tables S3 and S4 in Multimedia Appendices 4 and 5. Coverage is detailed in the results, with CIs for the coverage probabilities based on the binomial distribution of the *R* coverage indicators [38]. All simulations and analyses were conducted with R Statistical Software (R Foundation for Statistical Computing) [46].

### Ethical Considerations

This study was reviewed and approved by the institutional review boards at participating sites and under a reliance agreement between the CDC and the Westat institutional review board (FWA# FWA00005551, expiry date 10/13/2027, IRB project number 6201.08). This activity was reviewed by the CDC and was conducted consistent with applicable federal law and CDC policy (eg, 45 CFR part 46.102(l)(2), 21 CFR part 56; 42 USC §241(d); 5 USC §552a; 44 USC §3501). This activity was reviewed and approved as a research activity by one VISION site. This study presented minimal risk to participants because there was no interaction or intervention with patients; therefore, a waiver of informed consent was granted.

## Results

### Comparing Strategies for Estimating VE

**Figure 1:**
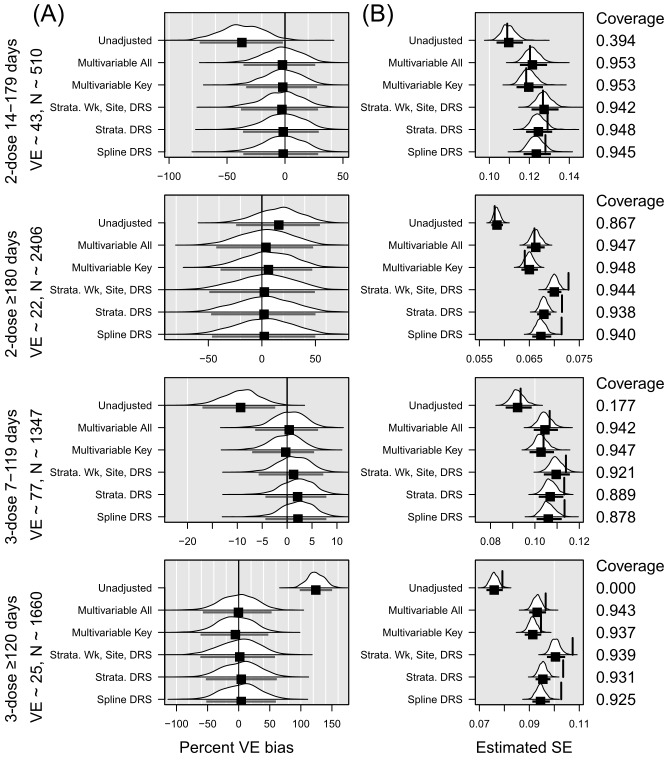
Summary of simulation results in the overall sample of 10,000. DRS: disease risk score; VE: vaccine effectiveness.

When N=10,000, the unadjusted models resulted in an average percent bias of VE ranging from –37% to 124% across the 4 vaccination categories (Figure 1, Table S3 in Multimedia Appendix 4). Average percent bias was dramatically lower in the models that accounted for the covariates: the 2 multivariable models without DRS (range for multivariable with all covariates, including key covariates from –2.2 to 3.8, multivariable with only key covariates from –5.3 to 6.1), the stratified model with DRS decile epi-week and site (range –2.6 to 2.3), the stratified model with DRS centile (range –1.5 to 4.2), and the model with DRS as a spline (range –1.6 to 4.1). Bias varied less across the 5 strategies for estimating VE than across the 4 levels of vaccination status for which VE was estimated. For example, the bias in estimates of 3-dose VE within 120 days ranged from –10% to 10% approximately, whereas the bias in estimates of 2-dose VE within 180 days ranged from –50% to 40% approximately. The difference in bias between vaccination status levels is related to the different sample sizes expected in each vaccine status group and the expected increasing instability of the percent bias when VE is closer to 0 (ie, 5 units is a much greater percent of 25 than it is of 80).

In all figures, panel A presents the percent VE bias for each vaccination level, with a vertical black line at 0. The distribution across 1000 replications is shown for each model strategy. The mean percent VE bias, represented with a black square, with an interval indicating the observed 2.5th and 97.5th percentiles, appears below each distribution. Panel B presents the distribution across 1000 replications of the estimated standard error for each vaccination level. The mean standard error, represented with a black square, with an interval indicating the observed 2.5th and 97.5th percentiles appears below the distribution. The vertical line shows the empirical standard error (SE), or SD of the coefficients. Ideally, the vertical line aligns with the mean standard error. Means to the left of the empirical SE indicate underestimation of the SE. Coverage is shown on the right of Panel B. Coverage is defined as the proportion of confidence intervals from the simulated analyses that include the true VE. Ideally, this would be close to 95%.

Across all 4 vaccination statuses, the multivariable models had the lowest SE. In most instances, the average SE came closest to the SD of the coefficients in the multivariable models. In all but the 2-dose 14-179 days category, the models incorporating DRS as a spline and the models stratified on DRS centile resulted in empirical SE greater than the 97.5th percentile of observed standard errors. The model stratified on decile of DRS, epi-week, and site fared slightly better, with empirical SE greater than the 97.5th percentile in 2 of the 4 vaccination categories. However, the models stratified on decile of DRS, epi-week, and site had the highest mean SE in all vaccination categories. Models including DRS as a spline had the greatest underestimation of SE, as demonstrated with the lowest ratio of mean SE to empirical SE (Table S3 in Multimedia Appendix 4). With the exception of the multivariable all or multivariate key models, all models yielded CIs that covered the true VE in less than 95% of replicated analyses for at least one vaccination status, as shown in Table 3.

**Figure 2:**
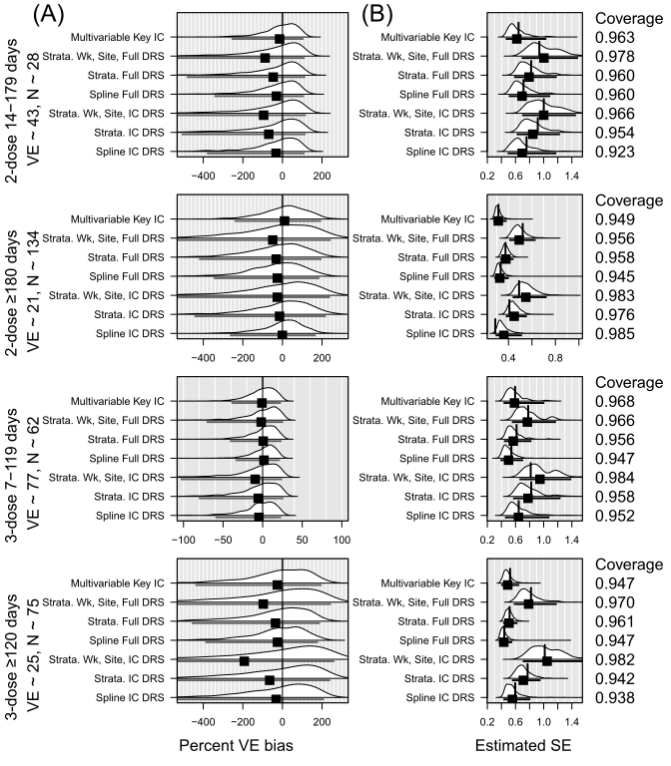
Summary of simulation results in the immunocompromised subset of 10,000. DRS: disease risk score; IC: immunocompromised; VE: vaccine effectiveness.

**Figure 3:**
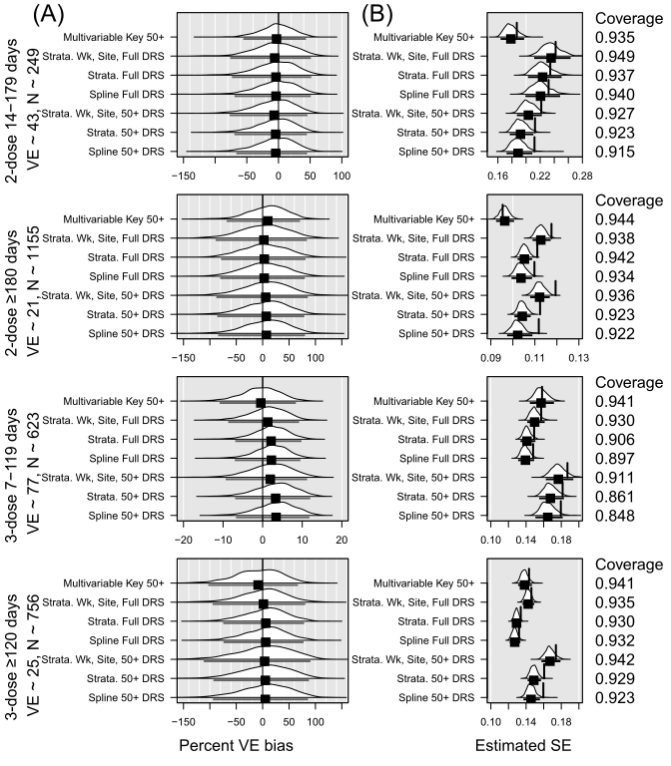
Summary of simulation results in the 50 years or older subset of 10,000. DRS: disease risk score; VE: vaccine effectiveness.

We also considered subgroups of the N=10,000 dataset defined by immunocompromised (IC) status and age, as these groups were routinely studied by VISION and others to guide vaccination policy decisions. Out of N=10,000, approximately 532 fell into the IC subgroup and 4782 into the 50 years or older subgroup. In the IC subgroup, the multivariable model with key covariates and without the DRS performed best in terms of bias—its VE estimates for each level of vaccination status were least biased (Figure 2 and Table S4 in Multimedia Appendix 5). This multivariable model also had the lowest SE while achieving 95% coverage across two of the four levels of vaccination status (Table 3). For estimates of 2-dose VE within 180 days, the coverage with the multivariable model was 96.8% (CI 95.1-97.5), and for 3-dose VE within 120 days, the coverage with the multivariable model was also 96.8% (CI 95.7-97.9). While having slightly higher bias, the spline model and the model stratified using DRS derived from the full 10,000 achieved 95% coverage in all 4 vaccination categories. The IC group was so small that several replications yielded very high SE estimates for at least one vaccination status indicating that a meaningful VE estimate was not obtained. This occurred in 21 of 1000 replications for the multivariate model, 11-132 replications for the models using the DRS from the full model, and 21-230 replications for the models using the DRS from the IC subset. In the older subgroup, the multivariable model with key covariates and without DRS again performed best in terms of bias across all 4 vaccination statuses (Figure 3 and Table S4 in Multimedia Appendix 5). Again, the multivariable model often had the lowest SE, which was also closest to the SD of the coefficients. Only the multivariable model achieved 95% coverage for its VE estimates for all four levels of vaccination status. Only the model stratified on DRS (from the full cohort), epi-week, and site-region achieved 95% coverage for 3 of the 4 levels of vaccination status; the other models only achieved this for 2 or fewer levels of vaccination status (Table 3). In both the IC subgroup and the 50 years or older subgroup, the models using a DRS derived from the subgroup tended to perform worse than models with a DRS derived from the full sample of 10,000.

**Figure 4:**
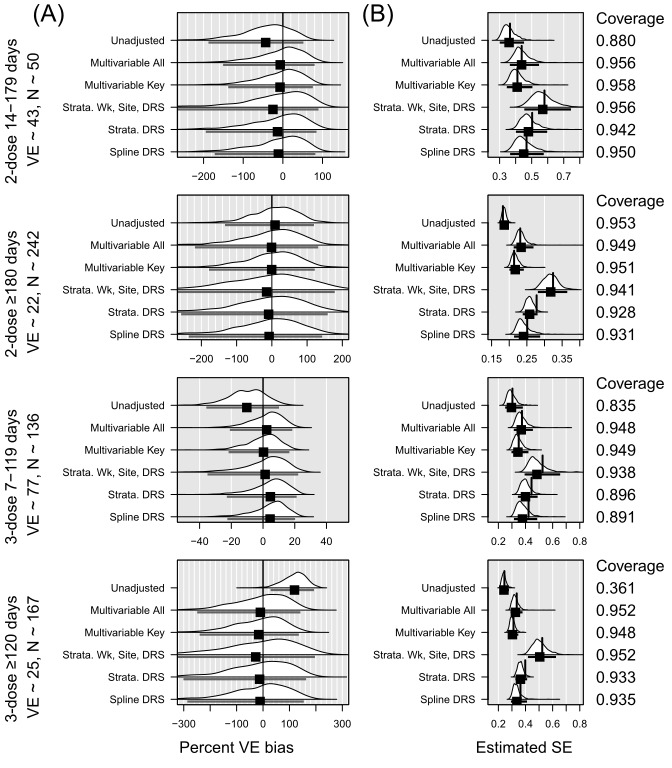
Summary of simulation results in a sample of size 1000. DRS: disease risk score; VE: vaccine effectiveness.

When N=1000, the sizes in each vaccination category were dramatically lower, ranging from 50 in the 2-dose 14-179 days group to 242 in the 2-dose ≥180 days group. Again, the multivariable models, particularly the one with all the covariates, had the least bias in VE estimates for each level of vaccination status (Figure 4 and Table S3). The multivariable models, along with the model stratified by epi-week, site, and DRS decile all achieved 95% coverage across the four vaccination statuses (Table 3). The model stratified by DRS centile and the model with a DRS spline had coverages slightly below 90% for 3-dose VE within 120 days (89.6% coverage CI: 87.7%-91.5% and 89.1% coverage CI: 87.2%-91%, respectively).

**Table 3:** Estimates of coverage of the true vaccine effectiveness (VE) with 95% CIs.

Model strategy		Two-dose 14-179 d, coverage^a^ (95% CI^b^)	Two-dose ≥180 d, coverage^a^ (95% CI^b^)	Three-dose 7-119 d, coverage^a^ (95% CI^b^)	Three-dose ≥120 d, coverage^a^ (95% CI^b^)
**N=10,000**					
	Multivariable all	95.3 (94.0-96.6)	94.7 (93.3-96.1)	94.2 (92.8-95.6)	94.3 (92.9-95.7)
	Multivariable key^c^	95.3 (94.0-96.6)	94.8 (93.4-96.2)	94.7 (93.3-96.1)	93.7 (92.2-95.2)
	Strata: week site, DRS^d^	94.2 (92.8-95.6)	94.4 (93.0-95.8)	92.1 (90.4-93.8)	93.9 (92.4-95.4)
	Strata: DRS	94.8 (93.4-96.2)	93.8 (92.3-95.3)	88.9 (87.0-90.8)	93.1 (91.5-94.7)
	Spline DRS	94.5 (93.1-95.9)	94.0 (92.5-95.5)	87.8 (85.8-89.8)	92.5 (90.9-94.1)
**IC^e^ subgroup**					
	Multivariable key^c^	96.3 (95.1-97.5)	94.9 (93.5-96.3)	96.8 (95.7-97.9)	94.7 (93.3-96.1)
	Strata: week site, DRS	97.8 (96.9-98.7)	95.6 (94.3-96.9)	96.6 (95.5-97.7)	97.0 (95.9-98.1)
	Strata: DRS	96.0 (94.8-97.2)	95.8 (94.6-97.0)	95.6 (94.3-96.9)	96.1 (94.9-97.3)
	Spline DRS	96.0 (94.8-97.2)	94.5 (93.1-95.9)	94.7 (93.3-96.1)	94.7 (93.3-96.1)
	Strata: week, site, IC DRS	96.6 (95.5-97.7)	98.3 (97.5-99.1)	98.4 (97.6-99.2)	98.2 (97.4-99.0)
	Strata: IC DRS	95.4 (94.1-96.7)	97.6 (96.7-98.5)	95.8 (94.6-97.0)	94.2 (92.8-95.6)
	Spline IC DRS	92.3 (90.6-94.0)	98.5 (97.7-99.3)	95.2 (93.9-96.5)	93.8 (92.3-95.3)
≥**50^f^ subgroup**					
	Multivariable key^c^	93.5 (92.0-95.0)	94.4 (93.0-95.8)	94.1 (92.6-95.6)	94.1 (92.6-95.6)
	Strata: week site, DRS	94.9 (93.5-96.3)	93.8 (92.3-95.3)	93.0 (91.4-94.6)	93.5 (92.0-95.0)
	Strata: DRS	93.7 (92.2-95.2)	94.2 (92.8-95.6)	90.6 (88.8-92.4)	93.0 (91.4-94.6)
	Spline DRS	94.0 (92.5-95.5)	93.4 (91.9-94.9)	89.7 (87.8-91.6)	93.2 (91.6-94.8)
	Strata: week, site, IC DRS	92.7 (91.1-94.3)	93.6 (92.1-95.1)	91.1 (89.3-92.9)	94.2 (92.8-95.6)
	Strata: IC DRS	92.3 (90.6-94.0)	92.3 (90.6-94.0)	86.1 (84.0-88.2)	92.9 (91.3-94.5)
	Spline IC DRS	91.5 (89.8-93.2)	92.2 (90.5-93.9)	84.8 (82.6-87.0)	92.3 (90.6-94.0)
**N=1000**					
	Multivariable all	95.6 (94.3-96.9)	94.9 (93.5-96.3)	94.8 (93.4-96.2)	95.2 (93.9-96.5)
	Multivariable key^c^	95.8 (94.6-97.0)	95.1 (93.8-96.4)	94.9 (93.5-96.3)	94.8 (93.4-96.2)
	Strata: week site, DRS	95.6 (94.3-96.9)	94.1 (92.6-95.6)	93.8 (92.3-95.3)	95.2 (93.9-96.5)
	Strata: DRS	94.2 (92.8-95.6)	92.8 (91.2-94.4)	89.6 (87.7-91.5)	93.3 (91.8-94.8)
	Spline DRS	95.0 (93.6-96.4)	93.1 (91.5-94.7)	89.1 (87.2-91.0)	93.5 (92.0-95.0)

^a^Coverage is defined as the proportion of confidence intervals from the simulated analyses that include the true VE. Ideally, this would be close to 95%.

^b^Confidence intervals were calculated using the properties of the binomial distribution of the indicators that each replication covers the true VE.

^c^Key variables include simple adjustment only for age (spline), epi-day (spline), and site region.

^d^DRS: disease risk score.

^e^IC: immunocompromising conditions.

^f^≥50: patients 50 years old or older.

### DRS-Related Decisions

When comparing the bias in analyses of subgroups defined by IC or age, we found little difference in the bias of VE between using a DRS built from the full dataset of 10,000 compared with using a DRS built exclusively from the subset of interest (Figures 2 and 3). When a difference was found, models using the DRS built from the full cohort tended to perform better than the models built from the smaller subset. Among the 12 VE estimates for the IC subgroup—a VE estimate for each of the 4 levels of vaccination status obtained by each of the 3 ways of using the DRS—9 of the 12 achieved 95% coverage when using a DRS from the full cohort compared with 5 of 12 when using the DRS from the IC subgroup. Among the 12 VE estimates for the subgroup aged ≥50 years, 6 of 12 achieved 95% coverage when using a DRS from the full cohort compared with 2 of 12 when using the DRS from the 50 years or older subgroup. Model-fitting challenges occurred more frequently when using the subgroup-specific DRS, particularly in the smaller IC subgroup.

## Discussion

### Principal Findings

This simulation study found that multivariable covariate adjustment, either with all the covariates or a subset of key covariates, performed well in the context of VISION’s case-control test-negative studies of VE. Adjustment for a DRS comprised of the covariates performed adequately but tended to overestimate the precision of VE estimates.

It is possible the underestimate of the SE we have observed could be attributed to a high correlation between the confounders and vaccination status. Early studies of DRSs suggested they can help reduce the dimensionality of analyses that try to adjust for multiple covariates but that DRS adjustment tends to overestimate the precision of the effect estimate of interest [47]. Later studies suggested that the corresponding exaggeration of the statistical significance of a finding (eg, a finding about VE) would be trivial unless the covariates are very strongly associated with the exposure (eg, vaccination status) or with each other [1,48].

It should not be surprising that simple covariate adjustment performed well, given VISION’s large samples of cases and controls. Other large VE studies with TNDs have also successfully employed individual covariate adjustment [35-37]. Furthermore, in previous VISION network studies, we observed that only a few of the available covariates (primarily age, calendar time, and geographic location) accounted for most of the confounding apparent in unadjusted VE estimates, which makes it feasible to adjust for the covariates individually even in subsets that might otherwise be too small to adjust for several dozen covariates or more. Even in our subgroup analyses, we did not find that use of the dimension-reducing DRS improved performance compared with straightforward adjustment for the individual covariates.

In the context of settings other than VISION, where sample sizes may be smaller, and the relevant covariates may be more numerous, the “curse of dimensionality” may render covariate adjustment more problematic. If an appropriate DRS has already been derived from large samples in similar settings, such a DRS could be helpful in several ways. First, an appropriate DRS can avoid overfitting. DRS adjustment outperformed individual covariate adjustment in some of our smaller subgroup analyses, especially when we used a DRS derived from the larger overall sample.

Second, confounding may arise from nonlinearities and interactions among covariate-outcome associations that could easily be overlooked unless previously captured by a DRS derived from large datasets using flexible machine learning methods. In this simulation study, we used boosted regression to derive the DRS, as described above, but our data-generating mechanism did not insert nonlinearities and interactions that would be undetectable in smaller datasets and would be challenging to specify in a logistic regression model with individual covariate adjustment.

Third, a DRS could be part of a hybrid approach to covariate adjustment. VE estimates could be derived from logistic regression models that include a DRS plus a few key covariates (which may also be components of the DRS). The key covariates would be those with high potential for confounding because of strong associations with the outcome (and exposure) that may differ in the current study population than in the population where the DRS was derived.

Fourth, a DRS can be an intuitive way to adjust for confounding and an intuitive tool to explore effect modification. Typically, effect modification examines one risk factor at a time—for example, examining whether the benefits of vaccination differ by age group. A DRS can be used to account for multiple risk factors as we examine whether the benefits of vaccination differ by level of risk. Furthermore, a DRS can facilitate the interpretation of findings. For example, if VE is found to be similar across levels of the DRS, yet the risk is 10-fold higher in the highest DRS decile as compared with the lowest, then we can infer that the vaccine benefits the highest decile 10-fold more (on the scale of cases prevented per 1000 persons vaccinated) than it benefits the lowest decile. However, a DRS may be less intuitive as a risk score in our test-negative VISION study than in a population-based cohort study to the extent that our test-negative controls are restricted to individuals with “a COVID-19-like illness” and may not be representative of the underlying population at risk. In the underlying population, risk factors for hospitalization for CLI may differ from risk factors for hospitalization for COVID-19.

This simulation study was motivated by challenges facing VISION’s studies of VE. The scenarios we simulated emulate those examined by VISION, and this may limit the generalizability of our findings to other settings.

### Conclusions

Our simulations found that logistic regression with individual covariate adjustment performed well in scenarios similar to those studied by the VISION network and is generally the current approach employed by the VISION network. DRS-adjusted models performed adequately but not as well as models that adjusted for the covariates individually.

## Appendix

Multimedia Appendix 1 Definition of effects of covariates on 13-level exposure, β, for multinomial model determining simulated exposure with exposure status defined by number of mRNA doses and time since most recent dose.

Multimedia Appendix 2 Definition of other coefficients, α, for the Bernoulli distribution defining the probability of the generated outcome.

Multimedia Appendix 3 Who is included in the disease risk score model?

Multimedia Appendix 4 Bias and standard error results, 5-level vaccine effectiveness, N=10,000 and 1000.

Multimedia Appendix 5 Bias and SE results, subgroup effect 5-level vaccine effectiveness.

## Abbreviations

CDC: Centers for Disease Control and Prevention
CLI: COVID-19–like illness
DRS: disease risk score
PS: propensity score
TND: test-negative design
VE: vaccine effectiveness
VISION: Virtual SARS-CoV-2, Influenza, and Other Respiratory Viruses Network

## Data Availability

The datasets generated during and/or analyzed during this study are not publicly available due to data sharing agreements between CDC and VISION network partner institutions prohibiting it.
