# Supplementary material for: Methods to Adjust for Confounding in Test-Negative Design COVID-19 Effectiveness Studies: Simulation Study
Source: JMIR Form Res. 2025 Jan 27;9:e58981. doi: 10.2196/58981 (PMC11811671; doi:10.2196/58981)
Supplement: Multimedia Appendix 1 [file formative_v9i1e58981_app1.docx]

**Table S1. Definition of effects of covariates on 13-level exposure,** $\boldsymbol{\beta}$**, for multinomial model determining simulated exposure with exposure status defined by number of mRNA doses and time since most recent dose**

|  | 2 doses, 14-59 days | 2 doses, 60-119 days | 2 doses, 120-179 days | 2 doses, 180-239 days | 2 doses, 240-299 days | 2 doses, 300-359 days | 2 doses, 360+ days | 3 doses, 7-59 days | 3 doses, 60-119 days | 3 doses, 120-179 days | 3 doses, 180-239 days | 3 doses, 240+ days |
| --- | --- | --- | --- | --- | --- | --- | --- | --- | --- | --- | --- | --- |
| (Intercept) | -4.60 | -3.04 | -3.36 | -1.65 | -2.13 | -3.36 | -14.58 | -3.47 | -4.53 | -7.42 | -12.64 | -13.33 |
| Epi-Day  (days since Jan 1, 2021) |  |  |  |  |  |  |  |  |  |  |  |  |
| Spline term 1 | 0.08 | -1.06 | 0.21 | -1.02 | -1.19 | 1.54 | 11.41 | -0.86 | 1.76 | 4.53 | 7.58 | 2.67 |
| Spline term 2 | -1.94 | -1.31 | -1.10 | -1.27 | -0.81 | -0.26 | 9.82 | -2.66 | -0.95 | 3.61 | 8.41 | 6.70 |
| Spline term 3 | -2.83 | -3.45 | -0.84 | -3.87 | -0.90 | -0.04 | 21.43 | -2.84 | -1.04 | 4.59 | 10.74 | 9.28 |
| Spline term 4 | -1.94 | -3.06 | -1.57 | -1.78 | -1.43 | -0.19 | 6.75 | -2.63 | -2.19 | 1.00 | 7.32 | 7.99 |
| Age |  |  |  |  |  |  |  |  |  |  |  |  |
| Spline term 1 | 0.05 | 0.29 | 0.51 | 0.55 | 0.91 | 0.68 | 0.84 | 1.25 | 1.25 | 1.28 | 1.52 | 2.40 |
| Spline term 2 | 0.05 | -0.46 | -0.33 | -0.28 | 0.56 | 1.28 | 2.10 | 1.62 | 3.00 | 3.42 | 4.13 | 5.71 |
| Spline term 3 | -0.19 | -0.30 | -0.57 | -1.18 | 0.42 | 1.96 | 3.19 | 2.05 | 3.44 | 3.73 | 5.22 | 7.87 |
| Spline term 4 | -0.48 | -0.32 | -0.60 | -1.26 | 0.54 | 1.98 | 2.86 | 1.76 | 2.09 | 2.31 | 2.77 | 2.43 |
| Sex (Male referent) | 0.35 | 0.40 | 0.34 | 0.26 | 0.25 | 0.32 | 0.29 | 0.34 | 0.29 | 0.28 | 0.27 | 0.24 |
| Race/Ethnicity  (Non-Hispanic White referent) |  |  |  |  |  |  |  |  |  |  |  |  |
| Hispanic | 0.24 | 0.25 | 0.2 | 0.18 | 0.01 | -0.05 | -0.26 | -0.15 | -0.32 | -0.46 | -0.58 | -0.86 |
| Non-Hispanic Black | 0.31 | 0.28 | 0.26 | 0.02 | -0.26 | -0.42 | -0.64 | -0.57 | -0.71 | -0.84 | -1.06 | -1.14 |
| Non-Hispanic Other | 0.11 | 0.22 | 0.18 | 0.22 | 0.14 | 0.05 | -0.14 | 0.09 | 0 | -0.10 | -0.14 | -0.37 |
| Unknown | -0.09 | -0.10 | -0.01 | -0.09 | -0.16 | -0.22 | -0.34 | -0.16 | -0.33 | -0.35 | -0.44 | -0.64 |
|  |  |  |  |  |  |  |  |  |  |  |  |  |
| Urban-Rural Classification at Admitting Facility  (Large Central Metro referent) |  |  |  |  |  |  |  |  |  |  |  |  |
| Large Fringe Metro | -0.12 | -0.14 | -0.07 | -0.11 | 0.01 | 0.03 | 0.12 | -0.05 | -0.04 | 0.03 | 0.06 | 0.16 |
| Medium Metro | 0.14 | 0.09 | 0.17 | -0.02 | -0.14 | -0.05 | -0.07 | -0.22 | -0.17 | -0.29 | -0.29 | -0.50 |
| Small Metro | 0.15 | 0.01 | 0.12 | 0 | 0.11 | 0.17 | 0.14 | 0.03 | 0.05 | 0.04 | 0.12 | -0.20 |
| Micropolitan | -0.04 | -0.13 | -0.04 | -0.22 | -0.03 | 0 | -0.09 | 0.02 | -0.08 | -0.18 | -0.23 | -0.74 |
| Non-Core | -0.28 | -0.11 | -0.51 | -0.33 | -0.22 | -0.05 | -0.15 | -0.21 | -0.29 | -0.39 | -0.47 | -0.57 |
| Unknown | 0.62 | 0.97 | 1.04 | 0.80 | 1.00 | 1.12 | 1.16 | 1.26 | 1.24 | 1.12 | 1.19 | 1.24 |
| Underlying Medical Conditions from Discharge Codes |  |  |  |  |  |  |  |  |  |  |  |  |
| Any non-respiratory underlying medical condition | -0.13 | -0.09 | -0.04 | -0.21 | -0.28 | -0.28 | -0.23 | -0.24 | -0.33 | -0.33 | -0.40 | -0.42 |
| Any respiratory underlying medical condition | -0.06 | -0.12 | -0.13 | -0.25 | -0.08 | -0.05 | -0.06 | -0.09 | -0.12 | -0.06 | -0.04 | -0.01 |
| Immunosuppression | 0.02 | 0.02 | 0.05 | 0.11 | 0.16 | 0.22 | 0.20 | 0.23 | 0.22 | 0.32 | 0.39 | 0.50 |
| Clinical Obesity | 0.17 | 0.16 | 0.14 | 0.22 | 0.21 | 0.12 | 0.06 | 0.11 | 0.16 | 0.06 | 0.09 | 0.04 |
| Diabetes Type II | -0.05 | -0.02 | 0.04 | 0.15 | 0.06 | 0.14 | 0.10 | 0.08 | -0.01 | 0.03 | 0.04 | 0.07 |
| Other Metabolic Disease | 0.18 | 0.25 | 0.13 | 0.12 | 0.10 | 0.15 | 0.21 | 0.17 | 0.26 | 0.25 | 0.34 | 0.41 |
| Renal Disease | 0.26 | 0.13 | 0.17 | 0.13 | 0.24 | 0.28 | 0.38 | 0.25 | 0.32 | 0.36 | 0.46 | 0.58 |
| Neurological/Musculoskeletal Disorder | -0.19 | -0.05 | -0.07 | -0.1 | -0.05 | -0.04 | -0.03 | -0.14 | -0.21 | -0.23 | -0.24 | -0.27 |
| Asthma | 0.28 | 0.31 | 0.26 | 0.36 | 0.25 | 0.10 | 0.13 | 0.26 | 0.28 | 0.19 | 0.20 | 0.11 |
| Other Chronic Lung Disease | -0.47 | -0.26 | -0.26 | -0.11 | -0.25 | -0.33 | -0.25 | -0.45 | -0.37 | -0.26 | -0.18 | -0.20 |
| Hypertension | 0.03 | 0.02 | 0.02 | 0.08 | 0.16 | 0.10 | 0.03 | 0.09 | 0.10 | 0.07 | 0.05 | -0.04 |
| COPD | 0.39 | 0.29 | 0.27 | 0.29 | 0.11 | 0.07 | -0.07 | 0.13 | 0.02 | -0.15 | -0.16 | -0.18 |
| Heart Failure | 0.12 | 0.20 | 0.09 | 0.03 | 0.01 | -0.04 | -0.08 | -0.03 | 0 | -0.18 | -0.15 | -0.16 |
| Ischemic Heart Disease | 0.20 | 0.08 | 0.19 | 0 | -0.06 | -0.03 | -0.02 | -0.01 | -0.11 | -0.07 | -0.14 | -0.07 |
| Other Heart Disease | -0.03 | 0.07 | 0 | 0.10 | 0.06 | 0.13 | 0.06 | 0.08 | 0.05 | 0.08 | 0.04 | 0.10 |
| Site-Region |  |  |  |  |  |  |  |  |  |  |  |  |
| A | 1.42 | 1.11 | 0.75 | 0.87 | 0.87 | 0.81 | 0.58 | 1.76 | 1.72 | 1.62 | 1.43 | 1.13 |
| B | 1.40 | 0.99 | 0.73 | 0.85 | 0.56 | 0.54 | 0.35 | 1.62 | 1.52 | 1.46 | 1.44 | 1.18 |
| C | 0.51 | -0.9 | -1.22 | -0.34 | -0.76 | -0.81 | -0.75 | -0.51 | -0.34 | -0.33 | -0.47 | -0.73 |
| D | -0.12 | -0.45 | -0.43 | -0.11 | -0.56 | -0.50 | -0.90 | -0.01 | -0.07 | -0.34 | -0.32 | -0.71 |
| E | -0.40 | -0.52 | -0.45 | 0.05 | 0.13 | 0.33 | 0.51 | 0.75 | 0.91 | 0.85 | 1.05 | 1.38 |
| F | -0.25 | -0.44 | -0.36 | 0.21 | 0.23 | 0.48 | 0.80 | 0.68 | 0.85 | 0.86 | 0.98 | 1.47 |
| G | -0.26 | -0.44 | -0.63 | -0.12 | -0.04 | 0.05 | 0.18 | 0 | 0.28 | 0.18 | 0.34 | 0.39 |
| H | 0.06 | -0.18 | -0.23 | -0.22 | -0.16 | 0.07 | 0.07 | -0.15 | -0.02 | 0.03 | -0.05 | -0.07 |
| I | 1.68 | 0.85 | 1.16 | 0.84 | 0.03 | 0.04 | -0.06 | 1.40 | 1.48 | 1.27 | 1.08 | 0.49 |
| J | 1.59 | 1.13 | 0.89 | 0.86 | 0.32 | 0.43 | 0.12 | 1.49 | 1.42 | 1.27 | 1.14 | 1.28 |
| K | 1.33 | 1.01 | 0.87 | 0.99 | 0.96 | 0.96 | 0.83 | 2.04 | 2.10 | 2.06 | 1.90 | 1.78 |
| L | 1.61 | 1.11 | 0.98 | 0.98 | 0.70 | 0.75 | 0.50 | 1.83 | 1.85 | 1.70 | 1.59 | 1.07 |
| M | 0.61 | -0.03 | -0.14 | 0.05 | -0.32 | -0.33 | -0.45 | 0.35 | 0.20 | -0.01 | -0.15 | -0.33 |
| N | 0.71 | 0.15 | 0.04 | 0.25 | -0.16 | -0.09 | -0.37 | 0.47 | 0.40 | 0.23 | 0.20 | 0.10 |
| O | 0.43 | 0.17 | 0.12 | 0.08 | -0.24 | -0.33 | -0.36 | 0.28 | 0.24 | 0.10 | 0.10 | -0.06 |
| P | 0.90 | 0.57 | 0.35 | 0.11 | -0.22 | -0.31 | -0.52 | 0.60 | 0.54 | 0.43 | 0.15 | -0.19 |
| Q | 0.92 | 0.62 | 0.54 | 1.10 | 0.41 | 0.48 | -0.13 | 1.67 | 1.74 | 1.57 | 1.32 | 0.61 |
| R | 0.75 | 0.92 | 0.91 | 1.18 | 0.61 | 0.38 | -0.06 | 1.49 | 1.62 | 1.48 | 1.33 | 0.67 |
| S | 1.40 | 1.15 | 1.08 | 1.35 | 0.93 | 0.87 | 0.54 | 2.17 | 2.11 | 2.19 | 1.89 | 1.60 |
| T | -0.25 | -0.93 | -0.96 | -0.45 | -0.97 | -1.03 | -1.32 | -0.40 | -0.50 | -0.59 | -0.83 | -1.37 |
| U | 1.29 | 0.99 | 0.75 | 0.57 | 0.36 | 0.31 | -0.08 | 1.39 | 1.20 | 1.00 | 0.63 | 0.18 |
| V | 1.65 | 1.06 | 0.89 | 1.32 | 0.66 | 0.66 | 0.40 | 2.14 | 2.12 | 1.99 | 1.78 | 0.78 |
| W | 1.76 | 0.99 | 0.82 | 1.19 | 0.74 | 0.78 | 0.49 | 2.30 | 2.28 | 2.16 | 2.01 | 1.25 |
|  |  |  |  |  |  |  |  |  |  |  |  |  |
