# Supplementary material for: Methods to Adjust for Confounding in Test-Negative Design COVID-19 Effectiveness Studies: Simulation Study
Source: JMIR Form Res. 2025 Jan 27;9:e58981. doi: 10.2196/58981 (PMC11811671; doi:10.2196/58981)
Supplement: Multimedia Appendix 2 [file formative_v9i1e58981_app2.docx]

**Table S2. Definition of other coefficients,** $\boldsymbol{\alpha}$**, for the Bernoulli distribution defining the probability of the generated outcome.**

|  | Coefficient |
| --- | --- |
| Intercept | -0.86 |
| 2 Doses, 14-59 days | -1.05 |
| 2 Doses, 60-119 days | -0.60 |
| 2 Doses, 120-179 days | -0.43 |
| 2 Doses, 180-239 days | -0.36 |
| 2 Doses, 240-299 days | -0.29 |
| 2 Doses, 300-359 days | -0.22 |
| 2 Doses, 360+ days | -0.16 |
| 3 Doses, 7-59 days | -1.61 |
| 3 Doses, 60-119 days | -1.39 |
| 3 Doses, 120-179 days | -0.43 |
| 3 Doses, 180-239 days | -0.16 |
| 3 Doses, 240+ days | -0.11 |
| Epi-Day (days since Jan 1, 2021) |  |
| Spline term 1 | -4.11 |
| Spline term 2 | -0.25 |
| Spline term 3 | 1.75 |
| Spline term 4 | -1.54 |
| Age |  |
| Spline term 1 | 0.40 |
| Spline term 2 | 0.36 |
| Spline term 3 | 0.79 |
| Spline term 4 | 0.54 |
| Sex (Male referent) | -0.07 |
| Race/Ethnicity  (Non-Hispanic White referent) |  |
| Hispanic | 0.14 |
| Non-Hispanic Black | 0.27 |
| Non-Hispanic Other | 0.23 |
| Unknown | 0.38 |
| Urban-Rural Classification at Admitting Facility  (Large Central Metro referent) |  |
| Large Fringe Metro | 0.15 |
| Medium Metro | 0.06 |
| Small Metro | 0.12 |
| Micropolitan | 0 |
| Non-Core | 0.07 |
| Unknown | 0.07 |
| Underlying Medical Conditions from Discharge Codes |  |
| Any non-respiratory underlying medical condition | -0.48 |
| Any respiratory underlying medical condition | -0.14 |
| Immunosuppression | -0.06 |
| Clinical Obesity | 0.15 |
| Diabetes Type II | 0.03 |
| Other Metabolic Disease | -0.06 |
| Renal Disease | 0.04 |
| Neurological/Musculoskeletal Disorder | -0.19 |
| Asthma | -0.07 |
| Other Chronic Lung Disease | 0.63 |
| Hypertension | 0 |
| COPD | -0.50 |
| Heart Failure | -0.48 |
| Ischemic Heart Disease | 0.04 |
| Other Heart Disease | -0.08 |
| Site-Region |  |
| A | -0.53 |
| B | -0.54 |
| C | -0.75 |
| D | -0.69 |
| E | -0.25 |
| F | -0.25 |
| G | -0.05 |
| H | 0.01 |
| I | -0.31 |
| J | -0.63 |
| K | -0.57 |
| L | -0.43 |
| M | -1.05 |
| N | -0.59 |
| O | -0.62 |
| P | 0.04 |
| Q | -0.13 |
| R | 0.14 |
| S | 0.21 |
| T | -0.18 |
| U | -0.50 |
| V | -0.17 |
| W | -0.21 |
