# Supplementary material for: Methods to Adjust for Confounding in Test-Negative Design COVID-19 Effectiveness Studies: Simulation Study
Source: JMIR Form Res. 2025 Jan 27;9:e58981. doi: 10.2196/58981 (PMC11811671; doi:10.2196/58981)
Supplement: Multimedia Appendix 3 [file formative_v9i1e58981_app3.docx]

### Who is included in the DRS Model?

The DRS has been described elsewhere [1-8]. For this simulation study, we used Miettinen’s approach which estimates the DRS from the entire study population and includes the exposure, in this case vaccination status, as part of the DRS model. Then, predicted DRS for the entire study population was generated assuming no one was treated [5]. Miettinen’s approach allows for a larger sample size to be used to estimate the DRS and performs comparably to a propensity score model and a direct covariate adjustment model as long as covariates are not too highly correlated with vaccination status [9], generally considered a correlation coefficient between confounders and exposure greater than 0.9 [3,4]. However, including exposure in the DRS limits its value as a balancing score. In Miettinen’s approach, incorrectly modeling the modification of exposure by baseline covariates can result in estimated scores that are influenced by the magnitude of the exposure effect [10]. Applied to estimating DRS, generalized boosted regression trees look for multilevel interactions, which are useful for describing the relationship between patient characteristics, including vaccination status, and the outcome. In contrast, the Peters and Belson approach to calculating the DRS, which uses only observations in the referent group, does yield a balancing score [11,13,14]. However, if DRS is strongly correlated with vaccination status, it still possesses bias concerns as in the Miettinen approach [9] and can be prone to over-fitting, particularly in the unvaccinated (referent) group [7]. If exposure is highly correlated with covariates, DRS with the full cohort is recommended [12]. Given the rapidly changing circumstances of the SARS-CoV-2 pandemic, using an out of sample model (either by time or location) to develop a DRS is not applicable here. Further application decisions are explored in our simulation study.

**References**

1. Arbogast PG, Kaltenbach L, Ding H, Ray WA. Adjustment for multiple cardiovascular risk factors using a summary risk score. Epidemiology. 2008:30-7. doi: 10.1097/EDE.0b013e31815be000.

2. Arbogast P, Seeger J, Group DMCSVW. Summary variables in observational research: propensity scores and disease risk scores. Effective health care program research report. 2012;33.

3. Arbogast PG, Ray WA. Use of disease risk scores in pharmacoepidemiologic studies. Statistical methods in medical research. 2009;18(1):67-80. doi: 10.1177/0962280208092347.

4. Arbogast PG, Ray WA. Performance of disease risk scores, propensity scores, and traditional multivariable outcome regression in the presence of multiple confounders. American journal of epidemiology. 2011;174(5):613-20. doi: 10.1093/aje/kwr143.

5. Miettinen OS. Stratification by a multivariate confounder score. American journal of epidemiology. 1976;104(6):609-20. doi: 10.1093/oxfordjournals.aje.a112339.

6. Tadrous M, Gagne JJ, Stürmer T, Cadarette SM. Disease risk score as a confounder summary method: systematic review and recommendations. Pharmacoepidemiology and drug safety. 2013;22(2):122-9. doi: 10.1002/pds.3377.

7. Wyss R, Glynn RJ, Gagne JJ. A review of disease risk scores and their application in pharmacoepidemiology. Current Epidemiology Reports. 2016;3(4):277-84. doi: 10.1007/s40471-016-0088-2.

8. Wyss R, Hansen BB, Ellis AR, Gagne JJ, Desai RJ, Glynn RJ, et al. The “dry-run” analysis: a method for evaluating risk scores for confounding control. American journal of epidemiology. 2017;185(9):842-52. doi: 10.1093/aje/kwx032.

9. Glynn RJ, Gagne JJ, Schneeweiss S. Role of disease risk scores in comparative effectiveness research with emerging therapies. Pharmacoepidemiology and drug safety. 2012;21:138-47. doi: 10.1002/pds.3231.

10. Hansen BB. The prognostic analogue of the propensity score. Biometrika. 2008;95(2):481-8. doi: 10.1093/biomet/asn004.

11. Cook EF, Goldman L. Performance of tests of significance based on stratification by a multivariate confounder score or by a propensity score. Journal of clinical epidemiology. 1989;42(4):317-24. doi: 10.1016/0895-4356(89)90036-x.

12. Tadrous M, Gagne JJ, Stürmer T, Cadarette SM. Disease risk score as a confounder summary method: systematic review and recommendations. Pharmacoepidemiology and drug safety. 2013;22(2):122-9. doi: 10.1002/pds.3377.

13. Peters CC. A method of matching groups for experiment with no loss of population. *The Journal of Educational Research*. 1941;34(8):606-612.

14. Belson WA. A technique for studying the effects of a television broadcast. *Journal of the Royal Statistical Society: Series C (Applied Statistics)*. 1956;5(3):195-202.
