# Supplementary material for: Methods to Adjust for Confounding in Test-Negative Design COVID-19 Effectiveness Studies: Simulation Study
Source: JMIR Form Res. 2025 Jan 27;9:e58981. doi: 10.2196/58981 (PMC11811671; doi:10.2196/58981)
Supplement: Multimedia Appendix 4 [file formative_v9i1e58981_app4.docx]

**Table S3. Bias and standard error results, 5-level VE, N=10000 and 1000**

| **N** | **Expo-sure** | **N Median and 95% Interval** | **True VE Mean and 95% Interval** | **Model** | **Percent Bias VE and 95% Confidence Interval** | **sd(**$\hat{\boldsymbol{\gamma}}$**)** | **mean** ${\hat{\boldsymbol{\sigma}}}_{\boldsymbol{\gamma}}$ **and 95% Confidence Interval** | **mean** ${\hat{\boldsymbol{\sigma}}}_{\boldsymbol{\gamma}}\boldsymbol{/}$**sd(**$\hat{\boldsymbol{\gamma}}$**)** |
| --- | --- | --- | --- | --- | --- | --- | --- | --- |
|  |  |  |  |  |  |  |  |  |
| **10,000** | **1** | **4078**  **(3985, 4204)** | **REF** |  |  |  |  |  |
|  | **2** | **510**  **(467, 555)** | **43.31**  **(42.33, 44.33)** | **Unadjusted** | -37.2 (-73.6, -1.8) | 0.109 | 0.110 (0.104, 0.117) | 1.007 |
|  |  |  |  | **Multivariable All** | -2.2 (-35.8, 26.1) | 0.120 | 0.122 (0.115, 0.129) | 1.010 |
|  |  |  |  | **Multivariable Key** | -1.9 (-33.5, 27.7) | 0.118 | 0.119 (0.114, 0.127) | 1.010 |
|  |  |  |  | **Strata. Week, Site, Full DRS** | -2.6 (-38.0, 28.6) | 0.127 | 0.127 (0.121, 0.134) | 1.004 |
|  |  |  |  | **Strata. Full DRS** | -1.5 (-36.2, 29.2) | 0.129 | 0.125 (0.118, 0.131) | 0.964 |
|  |  |  |  | **Spline** | -1.6 (-35.9, 28.6) | 0.128 | 0.123 (0.117, 0.131) | 0.964 |
|  | **3** | **2406**  **(2320, 2489)** | **21.79**  **(21.56, 22.01)** | **Unadjusted** | 15.8 (-24.0, 54.2) | 0.058 | 0.059 (0.057, 0.060) | 1.008 |
|  |  |  |  | **Multivariable All** | 3.8 (-42.6, 47.9) | 0.066 | 0.066 (0.065, 0.068) | 1.004 |
|  |  |  |  | **Multivariable Key** | 6.1 (-38.6, 47.1) | 0.064 | 0.065 (0.063, 0.067) | 1.014 |
|  |  |  |  | **Strata. Week, Site, Full DRS** | 2.3 (-49.1, 49.5) | 0.073 | 0.070 (0.069, 0.071) | 0.961 |
|  |  |  |  | **Strata. Full DRS** | 2.0 (-47.4, 50.1) | 0.072 | 0.068 (0.067, 0.069) | 0.949 |
|  |  |  |  | **Spline** | 2.3 (-46.6, 49.9) | 0.071 | 0.067 (0.066, 0.069) | 0.942 |
|  | **4** | **1347**  **(1275, 1415)** | **77.03**  **(76.89, 77.17)** | **Unadjusted** | -9.3 (-17.0, -2.4) | 0.094 | 0.092 (0.087, 0.099) | 0.985 |
|  |  |  |  | **Multivariable All** | 0.4 (-6.4, 6.2) | 0.107 | 0.105 (0.099, 0.110) | 0.980 |
|  |  |  |  | **Multivariable Key** | -0.3 (-7.0, 5.4) | 0.104 | 0.103 (0.098, 0.109) | 0.989 |
|  |  |  |  | **Strata. Week, Site, Full DRS** | 1.3 (-5.7, 7.3) | 0.114 | 0.110 (0.104, 0.116) | 0.961 |
|  |  |  |  | **Strata. Full DRS** | 2.1 (-4.4, 7.9) | 0.113 | 0.107 (0.102, 0.113) | 0.944 |
|  |  |  |  | **Spline** | 2.2 (-4.4, 7.9) | 0.113 | 0.106 (0.101, 0.112) | 0.935 |
|  | **5** | **1660**  **(1565, 1740)** | **25.46**  **(24.91, 25.98)** | **Unadjusted** | 124.1 (98.2, 150.3) | 0.079 | 0.076 (0.073, 0.079) | 0.958 |
|  |  |  |  | **Multivariable All** | -0.4 (-57.5, 53.0) | 0.097 | 0.093 (0.090, 0.097) | 0.965 |
|  |  |  |  | **Multivariable Key** | -5.3 (-61.4, 47.7) | 0.095 | 0.091 (0.088, 0.095) | 0.965 |
|  |  |  |  | **Strata. Week, Site, Full DRS** | 1.7 (-61.7, 58.5) | 0.107 | 0.100 (0.097, 0.104) | 0.936 |
|  |  |  |  | **Strata. Full DRS** | 4.2 (-52.8, 61.4) | 0.104 | 0.095 (0.092, 0.098) | 0.921 |
|  |  |  |  | **Spline** | 4.1 (-52.1, 59.9) | 0.103 | 0.094 (0.091, 0.098) | 0.919 |
| **1,000** | **1** | **405**  **(375, 436)** | **REF** |  |  |  |  |  |
|  | **2** | **50**  **(37, 65)** | **43.36**  **(40.21, 46.73)** | **Unadjusted** | -43.34 (-187.72, 51.87) | 0.365 | 0.359 (0.301, 0.452) | 0.985 |
|  |  |  |  | **Multivariable All** | -6.68 (-150.80, 80.09) | 0.438 | 0.437 (0.364, 0.546) | 0.998 |
|  |  |  |  | **Multivariable Key** | -7.14 (137.85, 75.67) | 0.411 | 0.410 (0.345, 0.505) | 0.996 |
|  |  |  |  | **Strata. Week, Site, Full DRS** | -24.99 (-277.22, 89.94) | 0.580 | 0.569 (0.454, 0.745) | 0.982 |
|  |  |  |  | **Strata. Full DRS** | -13.53 (-194.44, 84.91) | 0.503 | 0.480 (0.401, 0.597) | 0.954 |
|  |  |  |  | **Spline** | -11.39 (-171.87, 82.07) | 0.468 | 0.450 (0.366, 0.575) | 0.962 |
|  | **3** | **242**  **(214, 271)** | **21.80**  **(21.10, 22.54)** | **Unadjusted** | 9.11 (-132.88, 119.47) | 0.182 | 0.186 (0.176, 0.199) | 1.021 |
|  |  |  |  | **Multivariable All** | -0.90 (-218.39, 131.47) | 0.231 | 0.234 (0.213, 0.269) | 1.011 |
|  |  |  |  | **Multivariable Key** | -0.45 (-178.41, 121.82) | 0.214 | 0.217 (0.200, 0.241) | 1.015 |
|  |  |  |  | **Strata. Week, Site, Full DRS** | -14.83 (-303.55, 178.43) | 0.324 | 0.318 (0.281, 0.365) | 0.980 |
|  |  |  |  | **Strata. Full DRS** | -9.16 (-257.48, 158.48) | 0.278 | 0.258 (0.238, 0.281) | 0.929 |
|  |  |  |  | **Spline** | -7.74 (-235.72, 142.77) | 0.251 | 0.241 (0.214, 0.288) | 0.959 |
|  | **4** | **136**  **(114, 156)** | **77.03**  **(76.63, 77.48)** | **Unadjusted** | -10.23 (-35.75, 10.07) | 0.304 | 0.296 (0.250, 0.381) | 0.976 |
|  |  |  |  | **Multivariable All** | 2.30 (-20.93, 18.45) | 0.374 | 0.369 (0.314, 0.455) | 0.986 |
|  |  |  |  | **Multivariable Key** | 0.30 (-21.73, 16.61) | 0.350 | 0.344 (0.296, 0.422) | 0.983 |
|  |  |  |  | **Strata. Week, Site, Full DRS** | 1.32 (-35.04, 22.17) | 0.526 | 0.484 (0.393, 0.656) | 0.921 |
|  |  |  |  | **Strata. Full DRS** | 4.63 (-22.83, 21.20) | 0.444 | 0.400 (0.344, 0.489) | 0.900 |
|  |  |  |  | **Spline** | 4.45 (-22.51, 20.25) | 0.423 | 0.377 (0.315, 0.486) | 0.893 |
|  | **5** | **167**  **(146, 188)** | **25.48**  **(23.90, 27.06)** | **Unadjusted** | -10.11 (-249.12, 141.47) | 0.335 | 0.326 (0.291, 0.378) | 0.971 |
|  |  |  |  | **Multivariable All** | 119.02 (28.59, 192.88) | 0.244 | 0.241 (0.216, 0.278) | 0.986 |
|  |  |  |  | **Multivariable Key** | -16.45 (-239.99, 135.52) | 0.312 | 0.304 (0.272, 0.347) | 0.975 |
|  |  |  |  | **Strata. Week, Site, Full DRS** | -27.61 (-471.32, 196.32) | 0.523 | 0.505 (0.418, 0.625) | 0.965 |
|  |  |  |  | **Strata. Full DRS** | -13.00 (-301.15, 162.94) | 0.398 | 0.363 (0.326, 0.412) | 0.912 |
|  |  |  |  | **Spline** | -11.18 (-286.38, 154.22) | 0.363 | 0.336 (0.293, 0.411) | 0.927 |

Abbreviations:

50+ = patients 50 years old or older

DRS = disease risk score

IC = patients with immunocompromising conditions

VE = vaccine effectiveness.
