# Supplementary material for: Methods to Adjust for Confounding in Test-Negative Design COVID-19 Effectiveness Studies: Simulation Study
Source: JMIR Form Res. 2025 Jan 27;9:e58981. doi: 10.2196/58981 (PMC11811671; doi:10.2196/58981)
Supplement: Multimedia Appendix 5 [file formative_v9i1e58981_app5.docx]

**Table S4. Bias and standard error results, Subgroup Effect 5-level VE.**

| **Subset from 10000** | **Expo-sure** | **N in Subgroup Median and 95% Interval** | **True VE Mean and 95% Interval** | **Model** | | **Percent Bias VE** | | **sd(**$\hat{\boldsymbol{\gamma}}$**)** | **mean** ${\hat{\boldsymbol{\sigma}}}_{\boldsymbol{\gamma}}$ | **mean** ${\hat{\boldsymbol{\sigma}}}_{\boldsymbol{\gamma}}\boldsymbol{/}$**sd(**$\hat{\boldsymbol{\gamma}}$**)** |
| --- | --- | --- | --- | --- | --- | --- | --- | --- | --- | --- |
|  |  |  |  | |  | |  |  |  |  |
| **IC** |  |  |  | |  | |  |  |  |  |
|  | **1** | **233**  **(203, 264)** | **REF** | |  | |  |  |  |  |
|  | **2** | **28**  **(19, 39)** | **42.69**  **(38.53, 46.95)** | | **Multivariate Key** | | -14.40 (-257.54, 106.09) | 0.644 | 0.618 (0.461, 1.039) | 0.961 |
|  |  |  |  | | **Strata. Week, Site, Full DRS** | | -87.48 (-783.73, 112.54) | 0.938 | 1.003 (0.688, 1.489) | 1.069 |
|  |  |  |  | | **Strata. Full DRS** | | -46.64 (-483.19, 115.94) | 0.822 | 0.793 (0.580, 1.187) | 0.964 |
|  |  |  |  | | **Spline Full DRS** | | -30.28 (-343.49, 109.34) | 0.713 | 0.693 (0.504, 1.094) | 0.971 |
|  |  |  |  | | **Strata. Week, Site, IC Only DRS** | | -95.04 (-949.09, 118.63) | 1.002 | 1.000 (0.693, 1.458) | 0.998 |
|  |  |  |  | | **Strata. IC Only DRS** | | -69.13 (-508.91, 117.17) | 0.916 | 0.845 (0.617, 1.232) | 0.922 |
|  |  |  |  | | **Spline IC Only DRS** | | -32.30 (-381.64, 112.17) | 0.756 | 0.695 (0.487, 1.179) | 0.918 |
|  | **3** | **134**  **(112, 156)** | **21.00**  **(20.04, 21.97)** | | **Multivariate Key** | | 10.77 (-241.72, 195.86) | 0.314 | 0.313 (0.274, 0.385) | 0.997 |
|  |  |  |  | | **Strata. Week, Site, Full DRS** | | -49.14 (-652.38, 244.53) | 0.521 | 0.491 (0.407, 0.632) | 0.943 |
|  |  |  |  | | **Strata. Full DRS** | | -31.27 (-421.53, 197.48) | 0.375 | 0.378 (0.330, 0.444) | 1.009 |
|  |  |  |  | | **Spline Full DRS** | | -25.81 (-346.99, 186.82) | 0.334 | 0.326 (0.280, 0.403) | 0.977 |
|  |  |  |  | | **Strata. Week, Site, IC Only DRS** | | -25.94 (-550.40, 240.09) | 0.491 | 0.548 (0.436, 0.726) | 1.117 |
|  |  |  |  | | **Strata. IC Only DRS** | | -14.71 (-444.06, 218.86) | 0.408 | 0.449 (0.378, 0.557) | 1.100 |
|  |  |  |  | | **Spline IC Only DRS** | | -0.54 (-264.58, 168.10) | 0.288 | 0.361 (0.290, 0.516) | 1.253 |
|  | **4** | **62**  **(48, 77)** | **76.70**  **(76.18, 77.21)** | | **Multivariate Key** | | -0.89 (-39.49, 23.15) | 0.595 | 0.585 (0.429, 1.010) | 0.984 |
|  |  |  |  | | **Strata. Week, Site, Full DRS** | | -1.80 (-70.76, 26.00) | 0.780 | 0.768 (0.550, 1.175) | 0.985 |
|  |  |  |  | | **Strata. Full DRS** | | 0.73 (-41.42, 23.84) | 0.615 | 0.568 (0.437, 0.826) | 0.924 |
|  |  |  |  | | **Spline Full DRS** | | 1.64 (-34.17, 21.88) | 0.541 | 0.502 (0.386, 0.709) | 0.928 |
|  |  |  |  | | **Strata. Week, Site, IC Only DRS** | | -9.42 (-103.58, 24.68) | 0.816 | 0.947 (0.656, 1.392) | 1.161 |
|  |  |  |  | | **Strata. IC Only DRS** | | -5.56 (-80.71, 26.35) | 0.823 | 0.778 (0.566, 1.239) | 0.945 |
|  |  |  |  | | **Spline IC Only DRS** | | -4.66 (-59.84, 23.33) | 0.646 | 0.642 (0.450, 1.083) | 0.995 |
|  | **5** | **75**  **(59, 92)** | **25.27**  **(23.30, 27.25)** | | **Multivariate Key** | | -25.99 (-439.96, 198.12) | 0.523 | 0.490 (0.396, 0.652) | 0.937 |
|  |  |  |  | | **Strata. Week, Site, Full DRS** | | -96.84 (-1000.78, 245.23) | 0.820 | 0.786 (0.577, 1.187) | 0.959 |
|  |  |  |  | | **Strata. Full DRS** | | -34.89 (-458.03, 189.81) | 0.516 | 0.508 (0.424, 0.629) | 0.984 |
|  |  |  |  | | **Spline Full DRS** | | -24.88 (-389.16, 180.29) | 0.440 | 0.434 (0.364, 0.553) | 0.984 |
|  |  |  |  | | **Strata. Week, Site, IC Only DRS** | | -192.74 (-1947.00, 262.42) | 1.015 | 1.049 (0.702, 1.585) | 1.033 |
|  |  |  |  | | **Strata. IC Only DRS** | | -64.61 (-924.00, 241.30) | 0.772 | 0.711 (0.550, 0.955) | 0.921 |
|  |  |  |  | | **Spline IC Only DRS** | | -31.74 (-581.77, 209.60) | 0.596 | 0.556 (0.428, 0.810) | 0.933 |
| **50+** |  |  |  | |  | |  |  |  |  |
|  | **1** | **1999**  **(1921, 2094)** | **REF** | |  | |  |  |  |  |
|  | **2** | **249**  **(216, 282)** | **43.08**  **(41.43, 45.07)** | | **Multivariate Key** | | -3.07 (-55.43, 43.40) | 0.187 | 0.178 (0.164, 0.197) | 0.955 |
|  |  |  |  | | **Strata. Week, Site, Full DRS** | | -6.89 (-77.09, 45.70) | 0.222 | 0.203 (0.187, 0.222) | 0.917 |
|  |  |  |  | | **Strata. Full DRS** | | -4.43 (-69.56, 44.83) | 0.213 | 0.192 (0.177, 0.210) | 0.903 |
|  |  |  |  | | **Spline Full DRS** | | -4.34 (-66.47, 45.10) | 0.212 | 0.189 (0.172, 0.209) | 0.890 |
|  |  |  |  | | **Strata. Week, Site, 50+ Only DRS** | | -6.01 (-76.26, 51.10) | 0.242 | 0.236 (0.211, 0.263) | 0.974 |
|  |  |  |  | | **Strata. 50+ Only DRS** | | -3.94 (-73.48, 51.69) | 0.234 | 0.223 (0.203, 0.250) | 0.954 |
|  |  |  |  | | **Spline 50+ Only DRS** | | -3.89 (-69.40, 50.99) | 0.232 | 0.221 (0.199, 0.248) | 0.951 |
|  | **3** | **1155**  **(1091, 1217)** | **20.86**  **(20.54, 21.17)** | | **Multivariate Key** | | 9.75 (-68.17, 70.71) | 0.095 | 0.096 (0.092, 0.101) | 1.010 |
|  |  |  |  | | **Strata. Week, Site, Full DRS** | | 5.87 (-87.75, 85.60) | 0.119 | 0.112 (0.108, 0.117) | 0.939 |
|  |  |  |  | | **Strata. Full DRS** | | 6.77 (-86.03, 78.95) | 0.112 | 0.104 (0.101, 0.108) | 0.928 |
|  |  |  |  | | **Spline Full DRS** | | 7.02 (-84.28, 80.40) | 0.112 | 0.102 (0.097, 0.109) | 0.914 |
|  |  |  |  | | **Strata. Week, Site, 50+ Only DRS** | | 2.31 (-88.97, 83.83) | 0.118 | 0.113 (0.109, 0.117) | 0.959 |
|  |  |  |  | | **Strata. 50+ Only DRS** | | 3.15 (-78.84, 81.26) | 0.111 | 0.105 (0.102, 0.109) | 0.947 |
|  |  |  |  | | **Spline 50+ Only DRS** | | 2.95 (-80.47, 79.92) | 0.110 | 0.104 (0.099, 0.109) | 0.945 |
|  | **4** | **623**  **(572, 677)** | **76.83**  **(76.68, 77.00)** | | **Multivariate Key** | | -0.46 (-10.86, 8.40) | 0.158 | 0.157 (0.144, 0.172) | 0.993 |
|  |  |  |  | | **Strata. Week, Site, Full DRS** | | 1.93 (-9.34, 11.24) | 0.187 | 0.177 (0.162, 0.194) | 0.945 |
|  |  |  |  | | **Strata. Full DRS** | | 3.28 (-6.95, 12.04) | 0.182 | 0.168 (0.155, 0.183) | 0.924 |
|  |  |  |  | | **Spline Full DRS** | | 3.40 (-6.73, 11.82) | 0.179 | 0.165 (0.151, 0.182) | 0.917 |
|  |  |  |  | | **Strata. Week, Site, 50+ Only DRS** | | 1.30 (-8.70, 9.19) | 0.157 | 0.150 (0.141, 0.161) | 0.952 |
|  |  |  |  | | **Strata. 50+ Only DRS** | | 2.13 (-6.93, 9.80) | 0.149 | 0.141 (0.134, 0.151) | 0.946 |
|  |  |  |  | | **Spline 50+ Only DRS** | | 2.21 (-7.04, 9.54) | 0.148 | 0.139 (0.131, 0.149) | 0.941 |
|  | **5** | **756**  **(682, 811)** | **25.20**  **(24.57, 25.85)** | | **Multivariate Key** | | -8.59 (102.94, 66.83) | 0.143 | 0.138 (0.131, 0.147) | 0.964 |
|  |  |  |  | | **Strata. Week, Site, Full DRS** | | 3.77 (-111.60, 90.56) | 0.174 | 0.167 (0.157, 0.179) | 0.961 |
|  |  |  |  | | **Strata. Full DRS** | | 5.12 (-93.21, 87.75) | 0.161 | 0.149 (0.142, 0.157) | 0.927 |
|  |  |  |  | | **Spline Full DRS** | | 5.07 (-94.16, 85.97) | 0.160 | 0.146 (0.137, 0.156) | 0.912 |
|  |  |  |  | | **Strata. Week, Site, 50+ Only DRS** | | 1.53 (-94.60, 80.79) | 0.146 | 0.142 (0.136, 0.149) | 0.975 |
|  |  |  |  | | **Strata. 50+ Only DRS** | | 5.97 (-74.61, 78.69) | 0.134 | 0.129 (0.125, 0.134) | 0.964 |
|  |  |  |  | | **Spline 50+ Only DRS** | | 5.83 (-74.05, 77.55) | 0.132 | 0.127 (0.122, 0.134) | 0.963 |

Abbreviations:

50+ = patients 50 years old or older

DRS = disease risk score

IC = patients with immunocompromising conditions

VE = vaccine effectiveness.
